# Exposure to Drinking Water Trihalomethanes and Their Association with Low Birth Weight and Small for Gestational Age in Genetically Susceptible Women 

**DOI:** 10.3390/ijerph9124470

**Published:** 2012-12-06

**Authors:** Asta Danileviciute, Regina Grazuleviciene, Jone Vencloviene, Algimantas Paulauskas, Mark J. Nieuwenhuijsen

**Affiliations:** 1 Department of Environmental sciences, Vytautas Magnus University, K. Donelaicio g. 58, LT-44248, Kaunas, Lithuania; E-Mails: a.danileviciute@gmf.vdu.lt (A.D.); j.vencloviene@gmf.vdu.lt (J.V.); a.paulauskas@gmf.vdu.lt (A.P.); 2 Centre for Research in Environmental Epidemiology (CREAL), Doctor Aiguader 88, 08003, Barcelona, Spain; E-Mail: mnieuwenhuijsen@creal.cat

**Keywords:** trihalomethanes, LBW, SGA, GSTT1, GSTM1

## Abstract

Little is known about genetic susceptibility to individual trihalomethanes (THM) in relation to adverse pregnancy outcomes. We conducted a nested case-control study of 682 pregnant women in Kaunas (Lithuania) and, using individual information on drinking water, ingestion, showering and bathing, and uptake factors of THMs in blood, estimated an internal THM dose. We used logistic regression to evaluate the relationship between internal THM dose, birth outcomes and individual and joint (modifying) effects of metabolic gene polymorphisms. THM exposure during entire pregnancy and specific trimesters slightly increased low birth weight (LBW) risk. When considering both THM exposure and maternal genotypes, the largest associations were found for third trimester among total THM (TTHM) and chloroform-exposed women with the *GSTM1–0* genotype (OR: 4.37; 95% CI: 1.36–14.08 and OR: 5.06; 95% CI: 1.50–17.05, respectively). A test of interaction between internal THM dose and *GSTM1–0* genotype suggested a modifying effect of exposure to chloroform and bromodichloromethane on LBW risk. However, the effect on small for gestational age (SGA) was not statistically significant. These data suggest that THM internal dose may affect foetal growth and that maternal GSTM1 genotype modifies the THM exposure effects on LBW.

## 1. Introduction

In the last decades, a number of epidemiologic studies have been carried out to determine the effect of water chlorination disinfection by-products (DBPs) on adverse pregnancy outcomes. However, there is little or no evidence for associations between total trihalomethane (TTHM) concentration and adverse birth outcomes relating to foetal growth and prematurity, with the possible exception of small for gestational age (SGA) [1,2,3]. Most of the previous investigations have evaluated crude THM exposure and these studies differed on control of maternal characteristics that could also to be associated with adverse pregnancy outcomes. Data from recent epidemiologic studies show that the health effects are related not only to THM levels, but also to water use habits, and various subject characteristics, among them genotype [4,5,6]. 

In human detoxification processes glutathione *S*-transferases (GSTs) catalyse the conjugation of glutathione to toxic compounds that may be excreted [7,8]. The polymorphic GST could be characterised as a class theta enzyme (GSTT1) by means of molecular biology. “Conjugator” and “non-conjugator” phenotypes are coincident with the presence (*GSTT1–1*) and absence (*GSTT1–0*) of the gene activity that may lead to altered individual susceptibility to environmental exposures [9,10]. 

To our knowledge, only a single study has considered the role of genetic polymorphisms of the genes *CYP2E1* and 5,10-methylenetetrahydrofolate reductase in the study of relationship between THMs constituents’ exposure and foetal growth [9]. Our case-control study sought to overcome several of the drawbacks of previous studies by using individual internal dose assessments based on detailed water use behaviours, individual THMs, and controlling for various confounding variables to examine relationships between the exposure and foetal growth in genetically susceptible women. The purpose of this study was to examine whether the polymorphisms of metabolic genes *GSTT1* and *GSTM1* affect the association of maternal exposure to THM with LBW and SGA risk. 

## 2. Experimental Section

### 2.1. Participants

This Kaunas (Lithuania) cohort study is as a part of European Commission FP6 Health Impacts of Long-term Exposure to Disinfection By-products in Drinking Water in Europe (HiWATE) project [11]. Details on study subjects and the methods have been reported elsewhere [12,13]. The subjects of this nested case-control study were 682 women, whose blood samples were collected for genetic analysis, and who delivered singleton live births. LBW was defined as an infant’s birth weight of less than 2,500 g. Infants were considered SGA if they were in the lowest 10th centile of birth weight for each gestational week stratified by infant gender and maternal ethnic group. The study ethics complied with the Declaration of Helsinki; and the women were enrolled in the study only if they consented to participate in the study. The research protocol was approved by the Lithuanian Bioethics Committee and informed consent was obtained from all subjects. 

### 2.2. Genotyping

DNA was purified from the peripheral blood using DNA purification kits (SORPOclean Genomic DNA Extraction Kit, Vilnius, Lithuania). The *GSTM1–* and *GSTT1–null* genotypes were identified by the multiplex polymerase chain reaction (PCR) as described by Arand* et al.* [14] to determine the presence (at least one allele present: AA or Aa) or absence (complete deletion of both alleles: aa) of *GSTM1* and *GSTT1* genes. The detailed method and PCR conditions can be found elsewhere [12]. 

### 2.3. Exposure Assessment

The target population was women living in Kaunas city’s four water treatment plant supply zones. However, the four water treatment plants, which disinfect ground water with sodium hypochlorite, produced different concentrations of THMs in finished water. One treatment plant (Petrasiunai) supplied finished water with higher levels of THMs (“high level THM site”, 54.9% subjects), and the three other plants supplied finished water with lower levels of all THMs (“low level THM site”) [13]. Water samples were collected four times per year over the 3-year study period (2007–2009). Samples were analysed at the University of the Aegean (Mitilini, Greece) using gas chromatography with electron capture detection [15]. Measurements included specific values for the four regulated THMs (chloroform, bromoform, bromodichloromethane, and dibromochloromethane) and nine haloacetic acids (HAAs). Selected samples were analyzed for five haloacetonitriles, two haloketones, chloropicrin, and chloral hydrate. In addition, selected samples were analyzed at the National Institute for Health and Welfare (THL, Helsinki, Finland) for the halogenated furanone MX. Only regulated THM data were evaluated in this study since the other halogenated DBPs were present only at low or sub μg/L levels, if detected at all. 

We used tap water THM concentration, derived as the average of quarterly sample values over the time that the pregnancy occurred from all sampling sites located in the each distribution system, and geocoded maternal address at birth to assign the individual women’s residential exposure index. Estimates of exposure index to total and specific THMs from drinking water were tabulated first as an average level at the tap over the pregnancy period [13]. Then THM levels in drinking water samples and personal information were combined. We calculated ingestion, showering, and bathing THM uptakes and added those up. The internal dose (amount of substance uptake through skin by contact, lungs by breathing and gastrointestinal tract by swallowing that is retained in body through different routes) was used as an exposure to THM index.

We combined every subject’s residential exposure index and water-use questionnaire data to assess individual exposure through ingestion of THMs. Women were asked to indicate the cup or glass size and number of cups or glasses of tap water consumed per day, including hot and cold beverages made from tap water. With this information, we calculated daily amounts of hot and cold tap water ingested. Integration of the information on residential THM levels (μg/L), ingested amounts (L/day), and modifications by heating using an estimated uptake factor of 0.00490 to derive an integrated index of blood concentration, expressed in micrograms per day (μg/d) [13,16,17].

We assumed a null THM level for any bottled water consumption since in local bottled water production chlorination and ozonation are not used. Finally, we addressed dermal absorption and inhalation by considering showering and bathing alone and combined with ingestion. We multiplied residential THM levels (μg/L) by frequency and average duration of bathing or showering per day (min/day) and calculated each mother’s trimester-specific and entire pregnancy average daily uptake of THM internal dose (mg/d) [13,16,18]. Finally, we combined this information with THM uptake by ingestion, using an estimated uptake factor expressed in micrograms per day [19]. We then used average daily total uptakes from ingestion, showering, and bathing in our analysis as categorised variables by median of THM internal dose (μg/d) in different maternal genotypes subgroups. 

### 2.4. Statistical Analysis

We applied a Chi square test to estimate the univariate associations between maternal and environmental characteristic and proportions of LBW and SGA. We used multivariate logistic regression to evaluate the association between maternal exposure of THMs and risk of LBW and SGA, controlling for maternal characteristics; the adjusted ORs with 95% confidence intervals are presented. The maternal dose was used categorized as binary variable. 

For the LBW analyses data were adjusted for square gestational age, marital status, maternal education, maternal smoking, paternal smoking, alcohol consumption, body mass index, blood pressure, ethnic group, pregnancy history, infant gender, and birth year. For SGA analyses we adjusted for parity, marital status, maternal education, maternal smoking, body mass index, and birth year.

Then we investigated whether the association between maternal exposure to THM and birth outcomes was modified by maternal genotypes. The subgroups were defined by maternal genotype for *GSTT1* (present, absent) and *GSTM1* (present, absent) and maternal exposure to THM status during pregnancy (above median/below median). 

Subsequently, we tested for the interaction effect of maternal THM exposure, *GSTT1* and *GSTM1* with LBW and SGA by adding all the product terms in the regression models, while adjusting for potential confounders. Two-tailed statistical significance was evaluated by using a *p* value of 0.05. All statistical analyses were carried out using the SPSS software for Windows version 12.0.1.

## 3. Results and Discussion

### 3.1. Results

In this manuscript only regulated THMs data were evaluated since the other halogenated DBPs were present only at low or sub μg/L levels, if detected at all. The mean sum (and standard deviation) of the dihalogenated and trihalogenated HAAs for the high level site was 0.5 (0.7) and 0.3 (0.7) μg/L, respectively, whereas they were 0.3 (0.8) and 0.1 (0.2) μg/L, respectively, for the low level sites. The mean values of other individual halogenated DBPs (*i.e.*, haloacetonitriles, haloketones, chloropicrin, chloral hydrate, monohalogenated HAAs) were all less than 1.0 μg/L each for both high and low THM sites. MX was only measured once for the high level site and it was not detected, whereas it was measured three times at the low level sites and was 0.6–1.5 ng/L. Thus, only THM data were evaluated in this analysis, since there was a substantial difference in THM occurrence between high and low THM sites.

The mean TTHM level the three water treatment plants in the low level sites from was 1.3 µg/L and in the high level site (Petrasiunai) it was 21.9 µg/L. The individual total uptake of TTHM ranged between 0.0025 and 2.4040 µg/day, and the median was 0.1733 µg/day. The total gestational chloroform uptake ranged between 0.0013 and 2.1328 µg/day, and median was 0.1424 µg/day. Daily uptake of bromodichloromethane ranged between 0.0001 and 0.34 µg/day, median was 0.0280 µg/day, and for dibromochloromethane it ranged between 0 and 0.064 µg/day, median was 0.0026 µg/day. 

Our analysis included a total of 682 pregnant women; among them 48.4% were exposed to low level THM and 51.6% were exposed to high level THM (Table 1). Among these, 59 infants were classified as LBW and 96 as SGA. The proportion of LBW and SGA cases tended to be higher among women of high THM level site to compare to the low THM level sites. The women recruited were predominantly Lithuanian in ethnic origin (96.8%), did not smoke (89.6%), and during pregnancy did not use alcohol (93.8%). The mean age at enrolment was 28.8 years and the women tended to be highly educated (48.1% with a university degree). In general, mothers who were single, had underweight and normal weight, and had previous preterm history delivered a higher proportion of LBW. The characteristics that were statistically significant associated with SGA were young maternal age, single women, first infant, underweight and normal weight, and maternal disease. The prevalence of *GSTT1–0* genotype was 16.4%, while *GSTM1–0* was 48.7%. Among mothers who delivered LBW infants, the prevalence of *GSTT1–0* genotype was higher compared to controls (20.3% *vs.* 16.4%, respectively). Similar results were found for *GSTM1–0* genotype (57.6% *vs.* 47.9%, respectively). On the other hand, among mothers who delivered SGA infants, the prevalence of *GSTM1–0* genotype was higher compared to controls (49.0% * vs.* 47.9%, respectively). 

**Table 1:** Percent distribution of low birth weight and small for gestational age by maternal characteristics and *p* value of chi square.

Characteristic Risk factors		Total N (%)	Control N (%)	Low birth weight N (%)	Small for gestation age N (%)
Maternal age					
	<20 years	17 (2.5)	10 (1.8)	1 (1.7)	7 (7.3) ^a^
	20–29 years	402 (58.9)	330 (60.1)	29 (49.2)	51 (53.1)
	≥30 years	263 (38.6)	209 (38.1)	29 (49.2)	38 (39.6)
Marital status					
	Married	532 (78.0)	439 (80.0)	40 (67.8) ^a^	66 (68.8) ^a^
	Not married	150 (22.0)	110 (20.0)	19 (32.2)	30 (31.2)
Maternal smoking					
	Non-smoker	611 (89.6)	491 (89.4)	53 (89.8)	86 (89.6)
	Smoker (<1 cig/day)	71 (10.4)	58 (10.6)	6 (10.2)	10 (10.4)
Paternal smoking					
	No	327 (48.3)	264 (48.4)	26 (44.8)	44 (46.3)
	Yes (<1 cig/day)	350 (51.7)	282 (51.6)	32 (55.2)	51 (53.7)
Alcohol consumption					
	No	640 (93.8)	516 (94.0)	55 (93.2)	88 (91.7)
	Yes (<1 alc unit/day)	42 (6.2)	33 (6.0)	4 (6.8)	8 (8.3)
Blood pressure					
	<140/80 mm/Hg	581 (85.2)	470 (85.6)	49 (83.1)	78 (81.3)
	≥140 or ≥90 mm/Hg	101 (14.8)	79 (14.4)	10 (16.9)	18 (18.7)
Ethnic group					
	Lithuanian	660 (96.8)	531 (96.7)	57 (96.6)	92 (95.8)
	Other	22 (3.2)	18 (3.3)	2 (3.4)	4 (4.2)
Maternal education					
	primary school	58 (8.5)	43 (7.8)	9 (15.2)	11 (11.4)
	secondary school	296 (43.4)	242 (44.1)	23 (39.0)	38 (39.6)
	university degree	328 (48.1)	264 (48.1)	27 (45.8)	47 (49.0)
Parity					
	No child	319 (46.8)	249 (45.4)	26 (44.1)	54 (56.2) ^a^
	≥ child	363 (53.2)	300 (54.6)	33 (55.9)	42 (43.7)
Body mass index					
	<25 Underweight and normal	238 (35.0)	169 (30.8)	33 (55.9) ^a^	50 (52.1) ^a^
	25–30 Overweight	308 (45.1)	256 (46.6)	20 (33.9)	37 (38.5)
	>30 Obesity	136 (19.9)	124 (22.6)	6 (10.2)	9 (9.4)
Hazard work exposure ^b^					
	No	608 (89.1)	489 (89.1)	51 (83.6)	87 (90.6)
	Yes	74 (10.9)	60 (10.9)	10 (16.4)	9 (9.4)
Maternal disease ^c^					
	No	461 (67.6)	364 (66.3)	37 (62.7)	76 (79.2) ^a^
	Yes	221 (32.4)	185 (33.7)	22 (37.3)	20 (20.8)
Premature baby					
	No	654 (95.9)	508 (92.5)	53 (89.8)^a^	93 (96.9)
	Yes	28 (4.1)	41 (7.5)	6 (10.2)	3 (3.1)
THM exposure area					
	Low level	330 (48.4)	279 (50.8)	26 (44.1)	44 (45.8)
	High level	352 (51.6)	270 (49.2)	33 (55.9)	52 (54.2)
Infant gender					
	Male	364 (53.4)	297 (54.1)	27 (45.8)	49 (51.0)
	Female	318 (46.6)	252 (45.9)	32 (54.2)	47 (49.0)
Socioeconomic status ^d^					
	Low	255 (37.4)	202 (36.8)	20 (33.9)	39 (40.6)
	Medium	321 (47.1)	262 (47.7)	29 (49.2)	42 (43.8)
	High	106 (15.5)	85 (15.5)	10 (16.9)	15 (15.6)
*GSTT1*					
	GSTT1–1	570 (83.6)	459 (83.6)	47 (79.7)	81 (84.4)
	GSTT1–0	112 (16.4)	90 (16.4)	12 (20.3)	15 (15.6)
*GSTM1*					
	GSTM1–1	350 (51.3)	286 (52.1)	25 (42.4)	49 (51.0)
	GSTM1–0	332 (48.7)	263 (47.9)	34 (57.6)	47 (49.0)

^a^ *p* < 0.05; ^b^ Hazard work exposure (chemicals, dust, noise, hard work and other, this was a subjective assessment of each participant); ^c^ Maternal disease: diabetes, renal and urinary, sexual, respiratory, heart and vascular, congenital heart disease; ^d^ Socioeconomic status—Low (housekeeper, workwoman, student, jobless); Medium (salaried, farmer); High (manager, businesswoman).

Maternal exposure to TTHM and chloroform internal dose above the median during the entire pregnancy was associated with a slight increase in OR for LBW and SGA to compared to the reference below median group, after adjustment for potential confounding factors (Table 2). We observed a tendency of increasing LBW risk with increasing pregnancy duration for subjects exposed to TTHM and chloroform. During the third trimester, the odds ratios for LBW were 1.33, 95% CI: 0.62–2.87; and OR: 1.45, 95% CI: 0.67–3.13, respectively, for TTHM and chloroform. Similarly, third trimester TTHM and chloroform exposures increased slightly in risk for SGA (OR: 1.33, 95% CI: 0.84–2.13; and OR: 1.31, 95% CI: 0.82–2.08). For the DBCM and BDCM exposure we also observed slightly elevated odds ratios for LBW and SGA. 

**Table 2:** Low birth weight and small for gestation age adjusted odds (OR) ratios and 95% confidence intervals (CI) for trimester-specific and entire pregnancy exposure to internal dose THM.

THM exposure ^a^		Low birth weight	Small for gestation age
		OR ^b^ (95% CI)	OR ^c^ (95% CI)
TTHM ^d^			
	Entire pregnancy	1.27 (0.59–2.74)	1.32 (0.83–2.10)
	First trimester	1.03 (0.48–2.23)	1.20 (0.75–1.91)
	Second trimester	1.15 (0.53–2.47)	1.17 (0.74–1.86)
	Third trimester	1.33 (0.62–2.87)	1.33 (0.84–2.13)
Chloroform			
	Entire pregnancy	1.24 (0.57–2.68)	1.31 (0.82–20.9)
	First trimester	1.15 (0.54–2.48)	1.25 (0.78–1.99)
	Second trimester	1.29 (0.60–2.76)	1.23 (0.77–1.95)
	Third trimester	1.45 (0.67–3.13)	1.31 (0.82–2.08)
BDCM ^d^			
	Entire pregnancy	1.26 (0.58–2.72)	1.31 (0.82–2.09)
	First trimester	1.28 (0.59–2.76)	1.30 (0.82–2.08)
	Second trimester	1.26 (0.58–2.73)	1.25 (0.79–2.00)
	Third trimester	1.27 (0.59–2.76)	1.31 (0.82–2.09)
DBCM ^d^			
	Entire pregnancy	3.00 (0.34–27.0)	1.35 (0.80–2.29)
	First trimester	1.76 (0.66–4.69)	2.19 (1.20–3.99)
	Second trimester	1.46 (0.62–3.42)	1.40 (0.82–2.39)
	Third trimester	1.54 (0.65–3.63)	1.68 (0.97–2.89)

^a^ Reference group below median; ^b^ Adjusted for marital status, square gestational age, maternal education, maternal smoking, paternal smoking, alcohol consumption, body mass index, blood pressure, premature baby, infant gender, and birth year; ^c^ Adjusted for parity, maternal status, maternal education, maternal smoking, body mass index, birth year; ^d^ TTHM—total trihalomethane; DBCM—dibromochloromethane, BDCM—bromodichloromethane.

When the *GSTT1* genotype was considered, the association between exposure to THM and LBW differed by genotype: OR for LBW among women exposed to TTHM during the entire pregnancy was 1.19 (95% CI: 0.50–2.82) and 7.40 (95% CI: 0.13–409) for the present and absent genotypes, respectively. The findings were similar for chloroform: in carriers of *GSTT1–0* genotype exposure was associated with higher OR during entire pregnancy than in carriers of *GSTT1–1* genotype, OR: 7.48 (95% PI: 0.13–441) and 1.19 (95% PI: 0.50–2.28), respectively. Similar results were found for all three trimesters. The findings were similar when the DBCM exposures were analyzed (Table 3). 

In analysis of SGA, the largest association was found among women exposed for the third trimester. Women carrying *GSTT1–0* genotype exposed to TTHM OR for third trimester were 1.51, 95% CI: 0.43–5.29, while for *GSTT1–1* it were 1.22, 95% CI: 0.73–2.03; and for exposed to chloroform OR were 1.75, 95% CI: 0.50–6.10 and 1.18, 95% CI: 0.71–1.97, respectively, for absent and present genotype. However, a test of interaction did not show statistically significant results. 

**Table 3:** Low birth weight and small for gestational age adjusted odds ratios (OR) and 95% confidence intervals (CI) for gestational exposure to internal dose THMs according maternal polymorphisms in the *GSTT1* gene.

THM exposure ^a^	*GSTT1* genotype	N(%) ^b^	Entire pregnancy OR (95% CI)	First trimester OR (95% CI)	Second trimester OR (95% CI)	Third trimester OR (95% CI)
LBW ^c^						
TTHM ^d^	*GSTT1–1*	24 (8.5)	1.19 (0.50–2.82)	0.97 (0.41–2.28)	1.10 (0.47–2.59)	1.23 (0.52–2.90)
	*GSTT1–0*		7.40 (0.13–409)	7.48 (0.13–441)	7.48 (0.13–441)	7.30 (0.14–391)
		5 (12.2)				
	Interaction		1.24 (0.18–8.40)	1.42 (0.21–9.59)	1.26 (0.18–8.55)	1.19 (0.17–8.09)
CH ^d^	*GSTT1–1*	24 (8.4)	1.19 (0.50–2.82)	1.12 (0.48–2.63)	1.25 (0.53–2.92)	1.35 (0.57–3.20)
	*GSTT1–0*		7.48 (0.13–441)	7.48 (0.13–441)	7.48 (0.13–441)	7.30 (0.14–391)
		5 (11.9)				
	Interaction		1.14 (0.17–7.67)	1.25 (0.19–8.45)	1.21 (0.18–8.14)	1.18 (0.17–8.00)
BDCM ^d^	*GSTT1–1*	24 (8.7)	1.34 (0.57–3.16)	1.37 (0.58–3.23)	1.34 (0.57–3.16)	1.36 (0.58–3.22)
	*GSTT1–0*		0.89 (0.05–15.7)	0.89 (0.05–15.7)	0.89 (0.05–15.7)	0.89 (0.05–15.9)
		4 (10.0)				
	Interaction		0.62 (0.09–4.23)	0.61 (0.09–4.17)	0.63 (0.09–4.30)	0.61 (0.09–4.17)
DBCM ^d^	*GSTT1–1*	27 (14.8)	1.16 (0.10–13.1)	1.22 (0.38–3.91)	1.16 (0.43–3.13)	1.41 (0.54–3.70)
	*GSTT1–0*		56.1 (0.00–2×10^7^)	8.79 (0.21–377)	1.20 (0.06–25.3)	0.54 (0.02–12.51)
		5 (14.7)				
	Interaction		0.40 (0.06–2.77)	0.95 (0.14–6.68)	0.84 (0.12–5.96)	0.91 (0.13–6.26)
SGA ^e^						
TTHM ^d^	*GSTT1–1*	43 (15.1)	1.30 (0.78–2.17)	1.17 (0.70–1.94)	1.23 (0.68–1.88)	1.22 (0.73–2.03)
	*GSTT1–0*		1.04 (0.29–3.74)	0.99 (0.28–3.58)	0.99 (0.28–3.58)	1.51 (0.43–5.29)
		5 (12.2)				
	Interaction		0.82 (0.22–3.00)	0.90 (0.25–3.28)	0.91 (0.25–3.34)	1.22 (0.34–4.33)
CH ^d^	*GSTT1–1*	43 (15.1)	1.30 (0.78–2.17)	1.23 (0.74–2.06)	1.18 (0.71–1.98)	1.18 (0.71–1.97)
	*GSTT1–0*		0.99 (0.28–3.58)	0.99 (0.88–3.58)	1.15 (0.32–4.11)	1.75 (0.50–6.10)
		5 (11.9)				
	Interaction		0.80 (0.22–2.92)	0.86 (0.24–3.14)	0.94 (0.26–3.44)	1.35 (0.38–4.81)
BDCM ^d^	*GSTT1–1*	42 (15.2)	1.28 (0.77–2.14)	1.30 (0.78–2.18)	1.28 (0.77–2.14)	1.29 (0.77–2.15)
	*GSTT1–0*		1.03 (0.29–3.69)	1.04 (0.30–3.67)	0.72 (0.19–2.71)	1.03 (0.29–3.69)
		5 (12.5)				
	Interaction		0.83 (0.23–3.04)	0.75 (0.21–2.79)	0.61 (0.16–2.35)	0.82 (0.23–3.02)
DBCM ^d^	*GSTT1–1*	38 (20.9)	1.29 (0.71–2.34)	1.85 (0.93–3.67)	1.20 (0.65–2.20)	1.89 (1.01–3.54)
	*GSTT1–0*		1.43 (0.43–4.76)	3.79 (0.89–16.1)	2.36 (0.66–8.46)	1.04 (0.31–3.53)
		7 (20.6)				
	Interaction		1.35 (0.38–4.80)	1.61 (0.45–5.82)	2.09 (0.58–7.53)	0.98 (0.28–3.43)

^a ^Reference group below median; ^b^ The number of cases above median for entire pregnancy;^ c^ Adjusted for marital status, square gestational age, maternal education, maternal smoking, paternal smoking, alcohol consumption, body mass index, blood pressure, ethnic group, pregnancy history, infant gender, and birth year; ^d^ TTHM, total trihalomethane; CH—Chloroform, DBCM—dibromochloromethane, BDCM—bromodichloromethane;^ e^ Adjusted for parity, maternal status, maternal education, maternal smoking, body mass index, birth year.

Table 4 the shows association of maternal exposure to THMs above the internal dose median in different *GSTM1* genotypes with LBW and SGA. The findings suggest that woman carriers of *GSTM1–0* genotype and exposed to THM had an increased risk for LBW and SGA compared to woman carriers of *GSTM–1* genotype. 

The highest risk for LBW was found during the third trimester among woman exposed to TTHM (OR: 4.37, 95% CI: 1.36–14.08) and chloroform (OR: 5.06, 95% CI: 1.50–17.05). Exposure to BDCM during the third trimester among woman carriers of *GSTM1–0* genotype was associated with OR: 1.43, 95% CI: 0.73–2.81 and exposure to DBCM produced OR: 1.55, 95% CI: 0.72–3.36. 

**Table 4:** Low birth weight and small for gestational age adjusted odds ratios (OR) and 95% confidence intervals (CI) for gestational exposure to internal dose THMs according maternal polymorphisms in the *GSTM1* gene.

THM exposure ^a^	*GSTM1* genotype	N(%) ^b^	Entire pregnancy OR (95% CI)	First trimester OR (95% CI)	Second trimester OR (95% CI)	Third trimester OR (95% CI)
LBW^ c^						
TTHM ^d^	*GSTM1–1*	8 (4.6)	0.34 (0.09–1.22)	0.32 (0.09–1.14)	0.34 (0.09–1.23)	0.34 (0.09–1.24)
	*GSTM1–0*		4.23 (1.25–14.32)^ e^	2.88 (0.90–9.22)	3.21 (1.01–10.2) ^e^	4.37 (1.36–14.08) ^e^
		21 (13.9)				
	Interaction		13.37 (2.36–75.8) ^e^	9.29 (1.71–50.35) ^e^	10.28 (1.88–56.23) ^e^	13.35 (2.41–73.87) ^e^
CH ^d^	*GSTM1–1*	8 (4.6)	0.34 (0.09–1.22)	0.43 (0.13–1.42)	0.48 (0.14–1.59)	0.35 (0.10–1.28)
	*GSTM1–0*		4.08 (1.20–13.9) ^e^	2.81 (0.87–9.03)	3.08 (0.96–9.87)	5.06 (1.50–17.05) ^e^
		21 (13.9)				
	Interaction		12.88 (2.27–73.2) ^e^	6.70 (1.29–34.73) ^e^	7.04 (1.34–37.0) ^e^	15.86 (2.75–91.40) ^e^
BDCM ^d^	*GSTM1–1*	9 (5.5)	0.55 (0.16–1.89)	0.57 (0.17–1.95)	0.56 (0.16–1.90)	0.55 (0.16–1.89)
	*GSTM1–0*		2.65 (0.85–8.23)	2.63 (0.85–8.14)	2.65 (0.85–8.23)	2.74 (0.88–8.51)
		19 (12.6)				
	Interaction		5.16 (1.01–26.52) ^e^	4.89 (0.96–25.0)	5.11 (1.00–26.24) ^e^	5.29 (1.03–27.15) ^e^
DBCM ^d^	*GSTM1–1*	12 (10.1)	0.94 (0.07–12.14)	2.52 (0.54–11.7)	0.74 (0.19–2.90)	1.36 (0.36–5.11)
	*GSTM1–0*		11.97 (0.42–337)	1.47 (0.41–5.34)	2.13 (0.67–6.81)	1.78 (0.55–5.75)
		20 (20.6)	13.75 (0.23–83.3)	0.88 (0.19–4.10)	3.05 (0.63–14.87)	1.95 (0.40–9.56)
	Interaction					
SGA ^f^						
TTHM ^d^	*GSTM1–1*	23 (13.2)	0.84 (0.42–1.68)	0.80 (0.40–1.61)	0.78 (0.39–1.57)	0.86 (0.43–1.74)
	*GSTM1–0*		1.80 (0.92–3.55)	1.60 (0.82–3.15)	1.54 (0.79–3.02)	1.81 (0.92–3.56)
		25 (16.6)				
	Interaction		2.26 (0.85–5.95)	2.18 (0.82–5.75)	2.07 (0.79–5.46)	2.19 (0.83–5.79)
CH ^d^	*GSTM1–1*	23 (13.2)	0.84 (0.42–1.68)	0.89 (0.45–1.78)	0.90 (0.45–1.80)	0.88 (0.44–1.78)
	*GSTM1–0*		1.78 (0.90–3.50)	1.59 (0.81–3.12)	1.52 (0.78–2.97)	1.74 (0.89–3.41)
		25 (16.3)				
	Interaction		2.12 (0.81–5.54)	1.87 (0.72–4.88)	1.71 (0.66–4.87)	1.98 (0.76–5.20)
BDCM ^d^	*GSTM1–1*	24 (14.5)	1.05 (0.52–2.10)	1.00 (0.50–2.01)	0.96 (0.48–1.93)	1.05 (0.52–2.10)
	*GSTM1–0*		1.42 (0.72–2.79)	1.50 (0.77–2.95)	1.42 (0.72–2.79)	1.43 (0.73–2.81)
		23 (15.2)				
	Interaction		1.42 (0.55–3.71)	1.60 (0.61–4.16)	1.55 (0.59–4.03)	1.42 (0.55–3.71)
DBCM ^d^	*GSTM1–1*	26 (21.8)	1.57 (0.72–3.40)	2.33 (0.91–5.95)	1.44 (0.67–3.12)	1.63 (0.73–3.64)
	*GSTM1–0*		1.09 (0.51–2.32)	1.74 (0.77–3.97)	1.23 (0.57–2.65)	1.55 (0.72–3.36)
		19 (19.6)				
	Interaction		0.61 (0.23–1.61)	0.43 (0.16–1.14)	0.58 (0.22–1.53)	0.87 (0.33–2.26)

^a^ Reference group below median; ^b^ The number of cases above median for entire pregnancy;^ c^ Adjusted for marital status, square gestational age, maternal education, maternal smoking, paternal smoking, alcohol consumption, body mass index, blood pressure, ethnic group, pregnancy history, infant gender, and birth year; ^d^ TTHM, total trihalomethane; CH—Chloroform, DBCM—dibromochloromethane, BDCM—bromodichloromethane; ^e^*p* < 0.05; ^f^ Adjusted for parity, maternal status, maternal education, maternal smoking, body mass index, birth year;

A test of interaction between maternal exposure to THM and maternal *GSTM1* genotypes shows statistically significant results for LBW of second and third trimesters for TTHM (OR: 10.28 and 13.35), chloroform (OR: 7.04 and 15.86), and BDCM (OR: 5.11 and 5.29) exposure. 

Adjusted analyses of SGA showed a consistent but small increase in ORs among woman carriers *GSTM1–0* genotype and exposed to THMs. In third trimester OR was 2.19, 95% CI: 0.83–5.79 for TTHM, OR: 1.98, 95% CI: 0.76–5.20 for chloroform, and OR: 1.43, 95% CI: 0.73–2.81 for BDCM exposure. These increases were more pronounced when the interaction was examined. However, a test of interaction between *GSTM1* genotypes and exposure to THMs didn’t show statistically significant results for SGA.

### 3.2. Discussion

In this study we examined the effects of THM exposure as internal dose on LBW and SGA. This study used a case-control design to analyze the genetic effects and the gene-environment interaction controlling for major confounding variables. No statistically significant associations were observed between quantitative estimates of internal dose of THMs and birth outcomes. Our data suggest that the polymorphisms of the metabolic gene GSTM1 may have an effect on the association of maternal exposure to THM with LBW and SGA risk. When we considered both individual THM exposure and maternal genotypes, we were able to demonstrate a consistent, statistically significant effect on LBW associated with TTHM, chloroform and BDCM compared with unexposed women. The largest associations for LBW were found for third trimester among TTHM and chloroform exposed women with the GSTM1–0 genotype (OR: 4.37, 95% CI: 1.36–14.08 and OR: 5.06, 95% CI: 1.50–17.05, respectively). The results were more pronounced when interactions of genotype and THM exposure were examined. The adjusted ORs for TTHM were 15.86, 95% CI: 1.36–14.08; for chloroform ORs were 5.06, 95% CI: 1.50–17.05; and for BDCM it was 5.29, 95% CI: 1.03–27.15. We did find a non-significant elevated risk for SGA for those exposed to TTHMs during the three trimesters with highest ORs during the third trimester among women carriers of the GSTM1–0 genotype (OR: 1.81, 95% CI: 0.92–3.56) and GSTT1–0 genotype (OR: 1.51, 95% CI: 0.43–5.29). These data suggest that women with an absence of the enzyme activity appear to be susceptible to the adverse effects of THMs, such as increased risk of LBW. 

To date, no other published study has evaluated the risk for LBW or SGA and THM constituents as individual internal doses in association with *GSTT1* and *GSTM1* genotype polymorphism. Our results are consistent with previous studies, which suggested that exposure to THMs in the third trimester has a greater adverse effect on LBW and SGA than exposure early in pregnancy [20]. Some authors have presented that term LBW risk mostly increased during the second trimester (OR: 1.50, 95% CI: 1.07 to 2.10) [21]. Our results show that highest SGA risk associated with TTHM exposure was found during the third trimester (OR: 1.33, 95% CI: 0.84 to 2.13), while Wright *et al.* [22] found increased risk of SGA for second trimester (OR: 1.13; 95% CI: 1.03 to 1.24). Some study results provide no evidence of any increased risk of LBW, TLBW, and preterm delivery at the relatively low concentration of TTHMs [23,24]. Data of a Swedish study shows that exposure to sodium hypochlorite increase LBW (OR: 1.15, 95% CI: 1.05 to 1.26) [25]. Kramer *et al.* [26] concluded that chloroform concentrations greater than or equal to 10 micrograms/litre were associated with an increased risk for intrauterine growth retardation (OR: 1.8, 95% CI: 1.1 to 2.9). 

Relative to previous epidemiologic studies of this issue, this study has the advantage of seeking to overcome the exposure assessment drawbacks by using individual internal dose estimation based on different routes, detailed water use behaviours and studying individual THM, to examine relationships between the exposure and foetal growth in genetically susceptible women. The major strength of our study is the concurrent measurement of THM concentrations that we used for internal dose estimation, over the course of pregnancy. As a result, assignment of trimester average residential THM concentrations and estimation of individual THM uptake through drinking, showering and bathing should be more accurate than those used in previous studies. 

Another advantage of this study was the extensive control for confounding variables estimated for studying population. We estimated the association between THM internal dose levels and LBW controlling for gestational age, family status, gestational age, education, maternal and paternal smoking, alcohol consumption, body mass index, blood pressure, ethnic group, pregnancy history, infant gender, and birth year, for which we were able to adjust. Lack of information regarding the validity of the internal dose assessment models that we used is one of the limitations of this study. However, not all women exposed to THM during pregnancy have adverse reproductive outcomes, and several studies have suggested that potential reasons lie in genotoxicity, oxidative stress, disruption of foliate metabolism, [27] and maternal genetic susceptibility [28,29,30]. 

The single study which analyzed drinking water contaminants, foetal growth and *CYP2E1* genetic polymorphisms was conducted in Canada [8]. There, the adjusted odds ratio for intrauterine growth restriction associated with exposure to average TTHM above the 29.4 μg/L was 13.20 (95% CI: 1.19–146.72). These findings suggest that exposure to THM at the highest levels can affect foetal growth in genetically susceptible newborns.

The metabolism of environmental toxicants includes the several allelic variants of the polymorphic GST group whose show impaired enzyme activities and increase the susceptibility to both environmental xenobiotics and adverse birth outcomes [31,32]. *GSTM1* polymorphism is found to be present in 40 to 60% of most populations. Among Kaunas pregnant women *GSTM1–0* genotype is present in 48.7% of subjects. The deficiency of *GSTM1* has been shown to increase DNA-adduct formation and cytogenic damage [33]. The frequency of the *GSTT1–0* allele has been reported to be 30 to 40% in Germany [34], while in Lithuania it is 16.4%. It is possible that GST induction represents part of an adaptive response mechanism to chemical stress [31], therefore genetic polymorphism of *GSTM1* and *GSTT1* may modify the oxidative stress caused by maternal exposure to THM and lead to adverse pregnancy outcomes. 

When the results of this study are interpreted, a few conditions should be considered. This is a low-risk population with low-level THM exposure and low prevalence of *GSTT1–null* genotype; these factors may limit the extrapolation of these results to other populations. The THM exposure classification was based on median internal dose level, and thus the possibility of bias in exposure classification exists. However, in this study, we controlled for the main variables that might confound the association between THM, genetic polymorphism and foetal growth; therefore, the residual confounding of the results by exposure is expected to be small. 

Previous studies have suggested several plausible gene-environment interaction explanations. First, chemical substances could disturb foetal and placental cellular regulation via elevated PAH-DNA adducts due to the increased activity of enzymes that metabolize toxins (e.g., *CYP1A1*) and lower or absent activity of enzymes that detoxify these compounds (e.g., *GSTT1–* and *GSTM1–null* genotypes) [35]. Second, gene-xenobiotic interactions may exert their synergistic effects through oxidative stress that occurs upon chemical exposure. In response to this stress various inflammatory cytokines are produced in lung tissue increasing inflammatory responses and immune responses [36]. Further, other environmental factors and genetic polymorphism of *GSTM1* and *GSTT1* may modify the response to oxidative stress and lead to adverse pregnancy outcomes [37]. 

Recently reported DBP toxicity of HIWATE program site water samples revealed that chronic mammalian cell cytotoxicity correlated highly with the numbers of DBPs identified and the levels of DBP chemical classes [38]. In this study we found that the number of identified DBPs, the level of DBPs, the cytotoxic potency, and the genotoxic potency were all higher for the Kaunas high level THM site samples *versus* low level THM site samples. This relationship supports the epidemiologic findings of this study for an association between adverse reproductive effects, exposure to DBPs and genetic sensibility. There was a clear difference in the genotoxic responses among the Kaunas high level THM site and low level THM sites and these data suggest a coherent association between the analytical chemistry, the* in vitro* toxicology, and the epidemiologic results of this study, although, the association observed between the internal THM dose, *GSTM1–0* genotype and LBW risk may be due to DBPs that were not studied, or other toxic water contaminants.

Since there have been only a single epidemiological study that included the association between GST gene polymorphism, human susceptibility to THMs and adverse birth outcomes, further study is required to clarify the role of the GST polymorphism in foetal development. Our results are preliminary and need to be confirmed in a larger sample with a greater contrast in THM concentrations and internal THM doses.

## 4. Conclusions

Our results add to a growing body of epidemiological research about the harmful effects of THM exposure on foetal development. We found evidence for an association between third-trimester and whole-pregnancy internal dose levels of THM and foetal growth in genetically susceptible women’s. Such as association, however, is modified by an individual’s genotype. Data on the interaction between THM exposures and GST genes polymorphism shows that effect of THM on LBW is amplified by the *GSTM1–0* genotype. Future research of the effects of DBPs on birth outcomes should include analyses by genetic susceptibility and address potential causes of variation in effect estimates.
